# Calcarides A–E, Antibacterial Macrocyclic and Linear Polyesters from a *Calcarisporium* Strain

**DOI:** 10.3390/md11093309

**Published:** 2013-08-29

**Authors:** Johanna Silber, Birgit Ohlendorf, Antje Labes, Arlette Erhard, Johannes F. Imhoff

**Affiliations:** Kiel Center for Marine Natural Products KiWiZ, GEOMAR Helmholtz Centre for Ocean Research Kiel, Am Kiel-Kanal 44, Kiel 24106, Germany; E-Mails: jsilber@geomar.de (J.S.); bohlendo@gmx.de (B.O.); alabes@geomar.de (A.L.); aerhard@geomar.de (A.E.)

**Keywords:** fungal natural products, *Calcarisporium* sp., calcarides A–E, 15G256 components, macrocyclic and linear polyesters, antibacterial activities, antibiotic, marine natural products

## Abstract

Bioactive compounds were detected in crude extracts of the fungus, *Calcarisporium* sp. KF525, which was isolated from German Wadden Sea water samples. Purification of the metabolites from the extracts yielded the five known polyesters, 15G256α, α-2, β, β-2 and π (**1**–**5**), and five new derivatives thereof, named calcarides A–E (**6**–**10**). The chemical structures of the isolated compounds were elucidated on the basis of one- and two-dimensional NMR spectroscopy supported by UV and HRESIMS data. The compounds exhibited inhibitory activities against *Staphylococcus epidermidis*, *Xanthomonas campestris* and *Propionibacterium acnes*. As the antibacterial activities were highly specific with regard to compound and test strain, a tight structure-activity relationship is assumed.

## 1. Introduction

**Figure 1:**
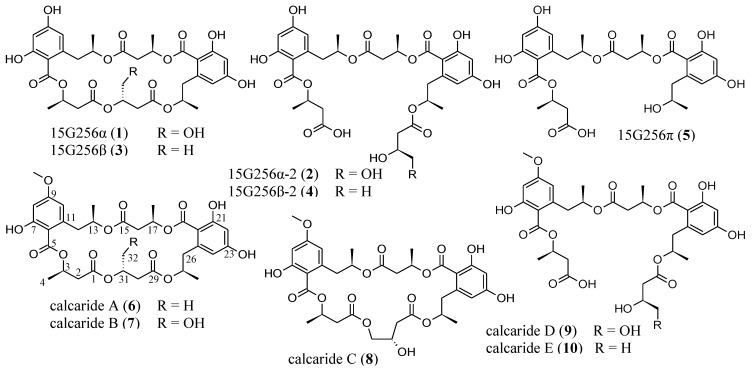
Compounds isolated from *Calcarisporium* sp. KF525: 15G256α, α-2, β, β-2 and π (**1**–**5**) and calcarides A–E (**6**–**10**).

Fungal species of the genus, *Calcarisporium*, have a widespread occurrence and are frequently found as mycoparasites or symbiotes of higher fungi [1,2,3,4,5]. The *Calcarisporium* sp. strain KF525 investigated in this study was isolated from a water sample taken in the German Wadden Sea. It showed a diverse chemical profile, including ten structurally closely related compounds of the 15G256-type known as, e.g., 15G256α (**1**) or α-2 (**2**) (Figure 1; to be differentiated from the structural class comprising 15G256γ, δ and ε).

**Figure 2:**
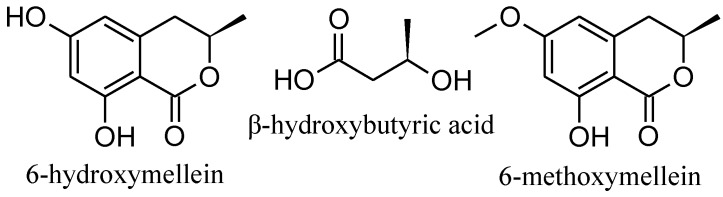
Proposed biosynthetic precursors of the calcarides (based on the proposal of Schlingmann *et al.* [6]).

The 15G256-type compounds are produced by a number of fungi, such as ascomycetes, like *Hypoxylon*, *Penicillium*, *Talaromyces*, *Acremonium* and *Scedosporium* species, as well as by the basidiomycete, *Albatrellus confluens* [6,7,8,9,10,11,12]. Since some metabolites were isolated independently from the different producer strains, renaming occurred among the 15G256-type compounds. 15G256α (**1**) is synonymous with BK233-A and NG-012, 15G256α-1 is a synonym of BK233-B and probably NG-011 (discussed in [6]), while 15G256β (**3**) was also named orbuticin, BK233-C and BE-26263 [6,7,9,10,13]. With regard to the chemical structures, the compounds are polyesters that occur either in closed ring structures as macrocycles or as linear molecules (Figure 1). Schlingmann *et al.* proposed a biosynthesis route in which 6-hydroxymellein and β-hydroxybutyric acid (Figure 2) are the precursors of the 15G256 components, forming the acyclic polyesters in the first instance. In later biosynthetic steps, the macrocyclic 15G256 compounds arise from the linear polyesters by ring closure reactions [6]. Various biological activities have been ascribed to the 15G256 agents, including antifungal, estrogenic and cytotoxic properties, as well as they were shown to potentiate nerve growth factor-induced neurite outgrowth [6,7,8,9,10]. For example, 15G256α (**1**), α-1 and β (**3**) attracted attention in the field of crop protection, as they displayed antifungal properties against important plant pathogenic fungi, e.g., *Botrytis cinerea* or *Monilinia fructigena* [7].

Here, we report the isolation of five known (**1**–**5**) and five new (**6**–**10**) 15G256-type compounds from *Calcarisporium* sp. strain KF525. Their structure elucidation and antibacterial activities are described.

## 2. Results and Discussion

### 2.1. Isolation and Structure Elucidation

The fungal strain KF525 is a *Calcarisporium* sp. that is a part of the marine fungal strain collection of the Kiel Center for Marine Natural Products [14]. Extracts of cultures from the *Calcarisporium* sp. strain KF525 grown in modified casamino acids glucose medium [15] showed antibacterial activities and were investigated in detail. Fractionation of these extracts by preparative reversed-phase HPLC yielded the known compounds, 15G256α, α-2, β, β-2 and π (**1**–**5**), as well as five new derivatives thereof, calcarides A–E (**6**–**10**) (Figure 1).

The structures of **1**–**5** were identified by comparison of their spectroscopic data (UV, MS and ^1^H NMR spectra) with those described in the literature [6]. As the data agreed well with the values published, the structures of **1**–**5** were assigned as shown in Figure 1.

The molecular formula of calcaride A (**6**) was determined to be C_33_H_40_O_14_ by high-resolution ESIMS measurements (measured 661.2503, calculated 661.2491 [M + H]^+^). The structure of **6** was elucidated on the basis of one- and two-dimensional NMR spectra (^1^H, ^13^C, DEPT, COSY, HSQC and HMBC, see Table 1). The NMR spectra of **6** were measured in acetone-*d*_6_, as it did not dissolve well in methanol-*d*_4_, whereas NMR data of all other calcarides were recorded in methanol-*d*_4_. The analysis of the NMR data of **6** revealed considerable structural similarities with 15G256β (**3**). The ^1^H NMR spectrum, the ^1^H NMR coupling constants and the ^13^C NMR spectrum of **6** showed that, comparable to **3** [6,7], the compound was composed of two phenyl moieties with their two protons in *meta* position to each other and five methyl, five methylene, five methine and five ester carbonyl groups. The two-dimensional NMR spectra of **6** proved that the aforementioned functional groups were connected exactly as in **3**, forming a cyclic pentalactone. A mass difference of 14 between **6** and **3** indicated the presence of an additional methyl group in **6**, which was corroborated by additional signals for a methyl group in the ^1^H and ^13^C NMR spectra. The chemical shifts of the respective proton and carbon resonances (δ_H_ 3.82 ppm, δ_C_ 55.8 ppm) determined it to be a methoxy group. The proton signal of the methoxy group correlated with C-9 in the HMBC NMR spectrum. Thus, the structure of **6** was identified as the methylated derivative of **3**, with the methyl group attached to the hydroxyl group of C-9.

**Table 1:** NMR spectroscopic data (500 MHz, acetone-*d*_6_) of calcaride A (**6**).

Position	δ_C_, Type	δ_H_, Mult. (*J* in Hz)	COSY	HMBC
1	170.09, C			
2a	40.6, CH_2_	2.91, dd (6.7, 16.3)	2b, 3	1, 3, 4
2b		2.74, dd (6.4, 16.3)	2a, 3	1, 3, 4
3	70.1, CH ^a^	5.56, m	2a, 2b, 4	1, 2, 4, 5
4	20.0, CH_3_	1.450, d (6.4)	3	1, 2, 3
5	171.0, C			
6	106.9, C			
7	165.5, C			
8	100.4, CH	6.36, d (2.6)	10	5, 6, 7, 9, 10
9	164.8, C			
OCH_3_	55.8, CH_3_	3.82, s		9
10	111.9, CH	6.44, d (2.6)	8	5, 6, 8, 9, 12
11	142.9, C			
12a	41.7, CH_2_	3.547, dd (6.8, 13.3)	12b, 13	6, 10, 11, 13, 14
12b		2.98, dd (7.5, 13.3)	12a, 13	6, 10, 11, 13, 14
13	73.1, CH	5.05, m ^b^	12a, 12b, 14	11, 12, 14, 15
14	19.7, CH_3_	1.19, d (6.2)	13	12, 13
15	170.12, C			
16a	41.02, CH_2_	2.90, dd (6.3, 15.8)	16b, 17	15, 17, 18
16b		2.72, dd (7.1, 15.8)	16a, 17	15, 17, 18
17	70.0, CH ^a^	5.52, m	16a, 16b, 18	15, 16, 18, 19
18	20.1, CH_3_	1.454, d (6.4)	17	15, 16, 17
19	170.9, C			
20	105.6, C			
21	165.9, C			
22	102.6, CH	6.30, d (2.5)	24	19, 20, 21, 23, 24
23	163.1, C			
24	113.3, CH	6.35, d (2.5)	22	19, 20, 22, 23, 26
25	143.3, C			
26a	42.4, CH_2_	3.553, dd (5.7, 12.9)	26b, 27	20, 24, 25, 27, 28
26b		2.78, dd (8.6, 12.9)	26a, 27	20, 24, 25, 27, 28
27	72.5, CH	5.01, m ^b^	26a, 26b, 28	25, 26, 28, 29
28	19.5, CH_3_	1.14, d (6.2)	27	26, 27
29	170.2, C			
30a	41.01, CH_2_	2.63 ^c^	30b, 31	29, 31, 32
30b		2.63 ^c^	30a, 31	29, 31, 32
31	68.4, CH	5.28, m	30a, 30b, 32	1, 29, 30, 32
32	19.9, CH_3_	1.27, d (6.3)	31	29, 30, 31

^a^ Assignments of C-3 and C-17 are interchangeable; ^b^ signals overlap and are deduced from the HSQC NMR spectrum; ^c^ signals overlap.

The molecular formula of calcaride B (**7**) was deduced to be C_33_H_40_O_15_ by high-resolution ESIMS measurements (measured 677.2435, calculated 677.2440 [M + H]^+^). UV and NMR spectra of **7** (Table 2, Table 3) strongly resembled those of **6**. The NMR spectra of **7** showed all resonances as those of **6**, apart from one striking difference: the signals in position 32 corresponded to a methylene instead of a methyl group. The downfield shift of the proton and carbon signals of CH_2_-32 (δ_H_ 3.65 and 3.58 ppm, δ_C_ 63.6 ppm) suggested the attachment of a hydroxyl group to C-32 in **7**. This was in full accordance with a mass increase of 16 mass units in comparison to **6** and determined **7** to be the methylated analog of the known compound, 15G256α (**1**).

**Table 2:** ^1^H NMR spectroscopic data of calcarides A–C (**6**–**8**) (500 MHz, acetone-*d*_6_ for **6**; 500 MHz, methanol-*d*_4_ for **7**–**8**).

	Calcaride A (6)	Calcaride B (7)	Calcaride C (8)
Position	δ_H_, Mult. (*J* in Hz)	δ_H_, Mult. (*J* in Hz)	δ_H_, Mult. (*J* in Hz)
2a	2.91, dd (6.7, 16.3)	2.88, dd (7.7, 16.6)	2.878, dd (8.1, 16.2)
2b	2.74, dd (6.4, 16.3)	2.73, d (5.3, 16.6)	2.79, dd (4.8, 16.2)
3	5.56, m	5.56, m	5.59, m
4	1.450, d (6.4)	1.42, d (6.3)	1.44, d (6.4)
8	6.36, d (2.6)	6.30, d (2.6)	6.31, d (2.6)
OCH_3_	3.82, s	3.75, s	3.74, s ^a^
10	6.44, d (2.6)	6.36, d (2.6)	6.35, d (2.6)
12a	3.547, dd (6.8, 13.3)	3.34 ^b^	3.35 ^c^
12b	2.98, dd (7.5, 13.3)	2.96, dd (6.4, 13.8)	2.99, dd (6.6, 14.0)
13	5.05, m ^d^	5.07, m	5.16, m
14	1.19, d (6.2)	1.20, d (6.2)	1.22, d (6.2)
16a	2.90, dd (6.3, 15.8)	2.81, dd (7.6, 15.8)	2.85, dd (6.6, 16.0)
16b	2.72, dd (7.1, 15.8)	2.67, dd (5.7, 15.8)	2.69, dd (6.6, 16.0)
17	5.52, m	5.49, m	5.48, m
18	1.454, d (6.4)	1.40, d (6.4)	1.39, d (6.3)
22	6.30, d (2.5)	6.22, s	6.21, d (2.5)
24	6.35, d (2.5)	6.22, s	6.23, d (2.5)
26a	3.553, dd (5.7, 12.9)	3.32 ^b^	3.31 ^c^
26b	2.78, dd (8.6, 12.9)	2.77, dd (8.1, 13.3)	2.885, dd (6.9, 13.4)
27	5.01, m ^d^	4.98, m	5.03, m
28	1.14, d (6.2)	1.11, d (6.2)	1.16, d (6.2)
30a	2.63 ^e^	2.68, dd (5.1, 16.7)	2.51, dd (6.0, 15.3)
30b	2.63 ^e^	2.64, dd (8.1, 16.7)	2.43, dd (7.2, 15.3)
31	5.28, m	5.28, m	4.17, m
32a	1.27, d (6.3)	3.65, dd (4.5, 11.9)	4.12, dd (4.8, 11.1)
32b		3.58, dd (5.1, 11.9)	4.08, dd (5.5, 11.1)

^a^ Position of the methoxy group could not unambiguously be determined. In analogy to the other calcarides, it was assumed to be linked to C-9. ^b^ Signal partially obscured and deduced from the HMBC NMR spectrum; ^c^ signal partially obscured; ^d^ signals overlap and are deduced from the HSQC NMR spectrum; ^e^ signals overlap.

**Figure 3:**
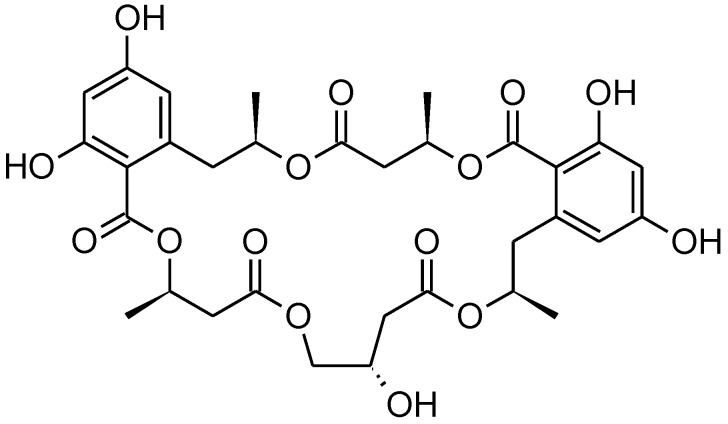
Structure of 15G256 α-1 [6].

Calcaride C (**8**) was found to have a molecular formula of C_33_H_40_O_15_ by means of high-resolution ESIMS measurements (measured 677.2441, calculated 677.2440 [M + H]^+^). Thus, **7** and **8** had the same molecular formula, showing that the closely related compounds were structural isomers, as was confirmed by comparison of the NMR spectra of the two compounds (Table 2, Table 3). For two of the known 15G256 components, 15G256α (**1**) and 15G256α-1 (Figure 3), a comparable regioisomerism was observed in which either β- or γ-hydroxyester linkages lead to the ring closure of the compound [6].

**Table 3:** ^13^C NMR shifts of calcarides A–E (**6**–**10**) (125 MHz, acetone-*d*_6_ for **6**; 125 MHz, methanol-*d*_4_ for **7**–**9**; 150 MHz, methanol-*d*_4_ for **10**).

	Calcaride A (6)	Calcaride B (7)	Calcaride C (8)	Calcaride D (9)	Calcaride E (10)
Position	δ_C_	δ_C_	δ_C_	δ_C_	δ_C_
1	170.09	171.5 ^a^	171.6	173.9 ^b^	175.9 ^b^
2	40.6	41.0	41.2	41.8	43.3
3	70.1 ^c^	70.2	70.5	70.8	71.5
4	20.0	20.2	20.2	20.1	20.3
5	171.0	171.13	171.53 ^d^	171.5 ^e^	171.56 ^f^
6	106.9	108.7	108.2	107.2	107.3
7	165.5	164.6	165.08 ^g,h^	166.0 ^i^	166.0 ^j^
8	100.4	100.7	100.7	100.8	100.8
9	164.8	164.9	165.0 ^h^	165.0 ^i^	165.0 ^j^
OCH_3_	55.8	55.8	55.8 ^k^	55.9	55.9
10	111.9	111.5	111.3	112.8	112.8
11	142.9	142.6	142.7	143.2	143.3
12	41.7	41.8	41.7	43.2	43.4
13	73.1	73.6	73.2	73.1	73.2
14	19.7	20.0	20.0	20.52	20.6
15	170.12	171.37 ^a^	171.3 ^d^	171.2	171.2
16	41.02	41.6	41.6 ^l^	41.8	41.8
17	70.0 ^c^	70.4	70.3	70.0	70.0
18	20.1	20.2	20.1	19.9	19.9
19	170.9	171.07	171.48 ^d^	171.6 ^e^	171.62 ^f^
20	105.6	106.7	107.3	105.8	105.7
21	165.9	165.4	165.14 ^g^	166.2	166.3
22	102.6	102.8	102.7	102.7	102.7
23	163.1	163.5	163.4	163.5	163.6
24	113.3	113.3	112.6	113.8	113.9
25	143.3	143.2	143.1	143.7	143.7
26	42.4	42.3	41.5 ^l^	43.5	43.6
27	72.5	73.5	73.6	72.7	72.6
28	19.5	19.5	19.8	20.49	20.6
29	170.2	171.43 ^a^	171.9	172.7	172.5
30	41.01	36.7	40.4	39.9	45.1
31	68.4	72.7	67.1	69.9	65.4
32	19.9	63.6	68.5	66.4	23.0

^a^, ^c–j^, ^l^ Assignments are interchangeable; ^b^ Signal deduced from the HMBC NMR spectrum; ^k^ Position of the methoxy group could not unambiguously be determined. In analogy to the other calcarides it was assumed to be linked to C-9.

Given that **7** is the methylated form of **1**, it was assumed that **8** was the methylated derivative of 15G256α-1. The NMR spectra of **8** confirmed the assumption, since the signals were in good agreement with those reported for 15G256α-1 [6]. An additional resonance for a methoxy group in the NMR spectra of **8** determined it to be the methyl ether of 15G256α-1. As the assignments of C-7 (δ_C_ 165.08 ppm) and C-9 (δ_C_ 165.0 ppm) might be interchangeable, the position of the methoxy group that showed a correlation to either of the two signals in the HMBC spectrum could not unambiguously be determined. In analogy to **6**, **7**, **9** and **10**, where the methyl group was linked to the hydroxyl group of C-9, it was deduced that this is also most likely to be the case for compound **8**. In addition to being regioisomers, it was observed that **7** and **8** interconverted during storage as dried compounds, with **7** showing a purity of about 75% after a period of two weeks. At the same time, compound **8** had converted into **7** more slowly and showed a purity of around 85%. These percentages were stable over a period of three months in which the compound stability was studied.

The molecular formula of calcaride D (**9**) was determined as C_33_H_42_O_16_ on the basis of high-resolution ESIMS measurements (measured 693.2405, calculated 693.2400 [M − H]^−^), requiring 13 degrees of unsaturation. The NMR spectra of **9** (Table 3, Table 4) showed signals for the two phenolic substructures characteristic of the calcarides and five carbonyl resonances, together accounting for 13 double bond equivalents. These data suggested a ring opening for **9**, giving the structure of a linear polyester, as has been found among the 15G256 components, as well, e.g., 15G256α-2 (**2**), β-2 (**4**) and π (**5**). MS and NMR spectra proved **9** to be the methylated analog of **2**. For the linear calcarides (**9** and **10**), the NMR spectra recorded in methanol-*d*_4_ gave one single signal for the aromatic protons of H-8 and H-10 (δ_H_ 6.33 ppm). Therefore, the position of the carbon signals of C-7 and C-9 (δ_C_ 166.0 and 165.0 ppm) could not be differentiated by the HMBC correlations of the aromatic protons resonances. As a consequence, the methoxy group showing a clear HMBC correlation to the signal at 165.0 ppm could have been attached to either C-7 or C-9. In order to determine the position of the methoxy group of **9**, additional NMR spectra were recorded in chloroform-*d*_1_ in which signals of H-8 and H-10 appeared separately. The HMBC NMR spectrum displayed correlations of H-8 to C-7 and C-9 and of H-10 to C-9 and C-12. Thereby, the positions of the signals for C-7 and C-9 could be assigned. Consequently, the methoxy group for which the HMBC NMR spectrum displayed a correlation to the resonance of C-9 had to be attached to C-9, as shown in Figure 1.

**Table 4:** ^1^H NMR spectroscopic data of calcarides D and E (**9** and **10**) (500 MHz, methanol-*d*_4_ for **9**; 600 MHZ, methanol-*d*_4_ for **10**).

	Calcaride D (9)	Calcaride E (10)
Position	δ_H_, Mult. (*J* in Hz)	δ_H_, Mult. (*J* in Hz)
2a	2.767, dd (7.5, 15.8)	2.72, dd (7.8, 15.4)
2b	2.69, dd (5.3, 15.8)	2.61, dd (5.9, 15.4) ^a^
3	5.57, m	5.58, m
4	1.43, d (6.4)	1.43, d (6.3)
8	6.33, s	6.33, s
OCH_3_	3.77, s	3.77, s
10	6.33, s	6.33, s
12a	3.29 ^b^	3.35, dd (3.5, 13.6) ^a^
12b	2.91, dd (9.7, 13.7)	2.87, dd (9.8, 13.6)
13	5.22, m	5.22, m
14	1.25, d (6.2)	1.26, d (6.3)
16a	2.66, dd (7.2, 15.7)	2.65, dd (7.2, 15.6)
16b	2.62, dd (5.8, 15.7)	2.62, dd (6.0, 15.6)
17	5.47, m	5.47, m
18	1.30, d (6.4)	1.30, d (6.3)
22	6.203, d (2.6)	6.19, d (2.5)
24	6.197, d (2.6)	6.18, d (2.5)
26a	3.17, dd (4.0, 13.4)	3.20, dd (3.8, 13.5)
26b	2.772, dd (9.2, 13.4)	2.74, dd (9.5, 13.5)
27	5.15, m	5.14, m
28	1.23, d (6.2)	1.23, d (6.2)
30a	2.47, dd (5.2, 15.4)	2.36, dd (6.9, 14.7)
30b	2.27, dd (7.9, 15.4)	2.26, dd (6.5, 14.7)
31	3.94, m	4.02, m
32a	3.44, dd (4.9, 11.2)	1.05, d (6.2)
32b	3.40, dd (5.7, 11.2)	

^a^ Signal partially obscured; ^b^ signal partially obscured and is deduced from the HSQC NMR spectrum.

The molecular formula of calcaride E (**10**) was determined to be C_33_H_42_O_15_ by high-resolution ESIMS measurements (measured 677.2467, calculated 677.2451 [M − H]^−^). The structure of **10** was readily deduced by comparing the NMR data (Table 3, Table 4) with those of **9**. In contrast to the hydroxylated methylene group at C-32 in **9**, the corresponding signals in the NMR spectra of **10** gave evidence for a methyl group not adjacent to oxygen. The position of the methoxy group at C-9 was determined according to additionally recorded NMR spectra in chloroform-*d*_1_, as described for **9**. Hence, the structure of **10** was established as the dehydroxylated form of **9**, which could equivalently be described as the methylated analog of **4**.

Because **6**–**10** were methylated forms of known compounds, it had to be ruled out that they were artifacts arising from the extraction protocol employing methanol as the solvent. The first indication that this was not the case for **6**–**10** was that the four demethylated analogs **1**–**4**, which were, as well, isolated from *Calcarisporium* sp. KF525, stayed demethylated when stored as methanolic solutions for time periods of more than two weeks. The final proof was that the isolation of **6**–**10** was likewise possible, when the use of methanol was avoided in all extraction steps and acetonitrile was employed instead. For this purpose, the presence of **6**–**10** was confirmed by using HPLC-UV/MS fingerprints of extracts from methanol and acetonitrile.

Absolute configurations of **6**–**10** were investigated by measurements of the optical rotation. The obtained values for optical activity were compared to those described for the demethylated analogs. The optical rotation data of **6**–**8** ([α]^22^_D_ −23.6, −25.3 and −7.9, respectively) were in good agreement with the reported ones of the corresponding demethylated forms, **3**, **1** and 15G256α-1 [6,7,8,9]. Even though some of the published values referred to ethanol solutions or higher temperatures employed for the measurements, they were consistent with our data. Hence, the absolute configurations of **6**–**8** were suggested to be the same as for their published demethylated forms [6]. For **2** and **4**, which are the demethylated derivatives of **9** and **10**, there is no literature record of optical rotation values. Thus, a comparison with published data was not possible, but as **2** and **4** were also isolated from *Calcarisporium* sp. KF525, their optical rotation was measured ([α]^22^_D_ −77.8 for **2**; [α]^22^_D_ −76.9 for **4**) and compared with the values of **9** and **10** ([α]^22^_D_ −71.8 and −67.3, respectively). As a result, the stereochemistry of **9** and **10** was assumed to be identical to the stereochemistry for **2** and **4** proposed by Schlingmann *et al.* [6] (Figure 1).

From a biosynthetic perspective, the precursors of the known 15G256 components are 6-hydroxymellein and β-hydroxybutyric acid [6]. Consequently, the building blocks of the calcarides should be 6-methoxymellein additional to 6-hydroxymellein and β-hydroxybutyric acid (Figure 2). 6-methoxymellein is a natural product known from a variety of fungi, e.g., *Aspergillus* and *Ceratocystis* species [16]. Both, 6-methoxy- and 6-hydroxy-mellein were found in crude extracts of *Calcarisporium* sp. KF525, according to UV and MS spectra from analytical HPLC-UV/MS analyses. The position of the methoxy and hydroxyl groups in the mellein structure could not be deduced from these data and was only assumed according to the position of the respective groups in the calcarides. Given that the calcarides are built up by the same or methylated precursors as the 15G256 components, the identical stereochemistry in calcaride and 15G256 molecules is reasonable.

### 2.2. Antibacterial Activity

Compounds **1**–**10** were profiled against a set of bacterial strains, including the biofilm-forming, *Staphylococcus epidermidis*, the plant pathogen, *Xanthomonas campestris*, and *Propionibacterium acnes*, which is linked to the skin disease acne. Even though the compounds are closely related from a structural point of view, they show distinct antibacterial activities (Table 5). All macrocyclic compounds (**1**, **3**, **6**–**8**) inhibited *S*. *epidermidis* and *X*. *campestris*, while the linear polyesters did not show any activities below a minimal inhibitory concentration (MIC) of 100 µM. The results indicated that a ring structure is required for the activity. This observation was also made for antifungal properties of the already known 15G256 components [6]. Among the cyclic compounds, **1** showed the highest activity against *S*. *epidermidis* ((12.9 ± 3.6) µM), and **6** displayed the lowest MIC for *X*. *campestris* ((5.5 ± 1.3) µM). A comparison of the inhibition between active methylated and demethylated forms, e.g. **1** and **7** or **3** and **6**, showed a decreased activity against *S*. *epidermidis* by a factor of four for the methylated analogs, whereas the activities concerning *X*. *campestris* were in the same concentration range. *P*. *acnes* was not inhibited by any of the tested substances, but the linear compound **5**. Given that **5** and **4** only differ in a β-hydroxybutyrate moiety, the *P*. *acnes*-related activity of **5** seemed highly specific.

**Table 5:** Antibacterial minimal inhibitory concentrations (MICs) of compounds **1**–**10** and the positive control, chloramphenicol. Activities higher than 20% were considered as inhibitory.

Compound	*S*. *epidermidis* MIC [µM]	*X*. *campestris* MIC [µM]	*P*. *acnes* MIC [µM]
15G256α (**1**)	12.9 (±3.6)	30.8 (±3.1)	>100
15G256α-2 (**2**)	>150	>150	>200
15G256β (**3**)	16.9 (±0.6)	14.9 (±7.5)	>200
16G256β-2 (**4**)	>150	>150	>100
15G256π (**5**)	>150	>150	14.1 (±1.8)
calcaride A (**6**)	68.8 (±3.7)	5.5 (±1.3)	>200
calcaride B (**7**)	52.3 (±2.3)	22.6 (±9.2)	>200
calcaride C (**8**)	29.6 (±1.6)	61.4 (±12.7)	>100
calcaride D (**9**)	>150	>150	>200
calcaride E (**10**)	104.3 (±7.8)	>150	>100
chloramphenicol	4.5	1.0	0.5

15G256 components are known to possess antifungal activities, estrogenic effects or cytotoxic properties with regard to cellular ATP (viability) and chemiluminescence (phagocytosis) in a mouse peritoneal macrophage assay, as well as they are described to be potentiators of the nerve growth factor in PC12 cells [6,7,8,9,10]. However, no antibacterial activities had been observed, even when tested. Here, the 15G256 compounds and the new methylated derivatives thereof were found to display antibacterial activities that are moderate in potency, but have a tight structure-activity relationship. Even a slight structural variation, like a methylation, as seen for the calcarides, influenced compound activities significantly. This was found to be true for other natural products [17,18]. For instance, the methylation of the cholesterol-lowering agent, lovastatin, resulted in the improved drug, simvastatin [19]. Regarding simvastatin, the introduction of a methyl group was used as a tool, but similarly, the isolation of derivatives of known compounds can be used as a strategy to obtain improved lead structures. As shown for the calcarides, fungi are a good source for this purpose, because they often produce a bunch of structurally related compounds, e.g., resulting from the promiscuity of the biosynthetic enzymes [20]. From the fungus’ point of view, the production of closely-related components might be of advantage as altered activities emerge.

## 3. Experimental Section

### 3.1. General Experimental Procedures

Optical rotation measurements were obtained on a Perkin Elmer model 241 polarimeter. UV spectra were measured on a Perkin Elmer Lambda 2 photometer. NMR spectra were recorded on a Bruker DRX 500 (500 and 125 MHz for ^1^H and ^13^C NMR, respectively) and a Bruker AV 600 spectrometer (600 and 150 MHz for ^1^H and ^13^C NMR, respectively). The residual solvent signals were used as internal references for NMR analyses (δ_H_ 3.31 and δ_C_ 49.0 ppm for methanol-*d*_4_; δ_H_ 2.05 and δ_C_ 29.8 ppm for acetone-*d*_6_, δ_H_ 7.26 and δ_C_ 77.2 ppm for chloroform-*d*_1_). High-resolution mass spectra were measured with a Bruker micrOTOF II, applying positive or negative ESI mode. Analytical reversed-phase HPLC-UV/MS experiments were conducted on a VWR-Hitachi LaChrom Elite system equipped with an L-2130 pump, an L-2450 diode array detector, an L-2200 autosampler, an L-2300 column oven and a Phenomenex Onyx Monolithic column (C18, 100 × 3.00 mm). A gradient with 0.1% formic acid in H_2_O (A) and 0.1% formic acid in acetonitrile (B) as solvents was applied: 0 min 5% B, 4 min 60% B, 6 min 100% B; flow 2 mL min^−1^. For mass detection, the analytical HPLC system was coupled to a Bruker esquire4000 ion-trap detector. Preparative HPLC was performed on a VWR LaPrep system consisting of a P110 pump, a P311 UV detector, a Labocol Vario-2000 fraction collector (LABOMATIC), a Smartline 3900 autosampler (Knauer) and a Phenomenex Gemini-NX column (10 μ C18, 100A, Axia, 100 × 50.00 mm). Subsequent compound purifications were performed on a Merck-Hitachi LaChrom Elite HPLC system comprised of an L-7150 pump, an L-2450 diode array detector and an L-2200 autosampler employing a reversed-phase Phenomenex Gemini-NX column (5 μ C18, 110A, Axia, 100 × 21.20 mm). 0.1% of formic acid in H_2_O (A) and 0.1% formic acid in acetonitrile (B) were the solvents used in preparative HPLC separations.

### 3.2. Fungal Material

*Calcarisporium* sp. KF525 is a part of the marine fungal strain collection of the Kiel Center for Marine Natural Products. The strain was cryo-conserved using the Microbank system (Pro-Lab).

### 3.3. Cultivation

*Calcarisporium* sp. KF525 was cultivated in 12 L of casamino acids glucose medium (0.25% casein hydrolysate, 4% glucose × H_2_O, 0.01% MgSO_4_ × 7H_2_O, 0.18% KH_2_PO_4_, pH 6.8) [15]. 2-L Erlenmeyer flasks filled with 0.75 L medium were used for the culture experiments, which were performed for 24 days at 22 °C under shaking conditions (120 rpm) in the dark. A circular agar slant (1.8 cm in diameter) of a preculture served as the inoculum. The preculture was grown on agar plates of modified Wickerham medium (0.3% malt extract, 0.3% yeast extract, 0.5% peptone from soymeal, 1% glucose × H_2_O, 3% NaCl, 1.5% agar, pH 6.25) [21] over a period of 11 days at room temperature in the dark.

### 3.4. Isolation Procedure

The cultures of *Calcarisporium* sp. KF525 were separated into mycelium and culture supernatant. An extraction of the culture supernatant with EtOAc yielded a crude extract of 0.92 g. The extract was subjected to the first purification step using a VWR LaPrep HPLC system (gradient: 0 min 10% B, 17 min 60% B, 22 min 100% B; flow 100 mL min^−1^; UV detection at 217 nm). The purification gave 8 fractions containing the 15G256 and calcaride components (*t*_R_ 12.4, 13.2, 14.5, 15.7, 16.2, 17.6, 18.5 and 20.6 min) of which fraction 8 comprised pure **6** in a yield of 17.7 mg. All other fractions were further purified on a Merck-Hitachi LaChrom Elite HPLC system applying reversed-phase chromatography. The purification of fraction 1 gave 17.8 mg of **2** (gradient: 0 min 30% B, 13 min 40% B; flow 18 mL min^−1^; UV detection at 220 nm; *t*_R_ 6.7 min) and fraction 2 afforded 11.8 mg of **5** (gradient: 0 min 25% B, 13 min 50% B; flow 18 mL min^−1^; UV detection at 220 nm; *t*_R_ 9.2 min). The chromatography of fraction 3 (gradient: 0 min 30% B, 15 min 37% B; flow 18 mL min^−1^; UV detection at 220 nm) resulted in 14 mg of purified **4** (*t*_R_ 11.1 min) and semi-pure **9** (*t*_R_ 12.0 min), which was isolated in two additional rounds of purification to give a yield of 23.2 mg (1st, gradient: 0 min 30% B, 13 min 45% B; flow 18 mL min^−1^; UV detection at 220 nm; *t*_R_ 9.2 min. 2nd, isocratic: 25% B; flow 18 mL min^−1^; UV detection at 210 nm; *t*_R_ 23.2 min). Pure **1** (17.5 mg) was obtained from fraction 4 by applying two consecutive steps of preparative HPLC (1st, 0 min 40% B, 13 min 65% B, flow 18 mL min^−1^; UV detection at 205 nm; *t*_R_ 5.2 min. 2nd, gradient: 0 min 30% B, 10 min 61% B; flow 18 mL min^−1^; UV detection at 220 nm; *t*_R_ 7.6 min). Fraction 5 was subjected to two further purification steps yielding 2.9 mg of **10** (1st, isocratic: 29% B, flow 18 mL min^−1^; UV detection at 220 nm; *t*_R_ 21.7 min. 2nd, gradient: 0 min 27% B, 15 min 50% B; flow 18 mL min^−1^; UV detection at 220 nm; *t*_R_ 12.4 min). Fraction 6 contained compounds **7** and **8**, which were initially eluted isocratically with 42% B (flow 18 mL min^−1^; UV detection at 220 nm; *t*_R_ 11.8 min). A subsequent round of preparative HPLC (isocratic: 40%, flow 18 mL min^−1^; UV detection at 210 nm) gave purified **7** (*t*_R_ 12.8 min) and **8** (*t*_R_ 16.0 min) in yields of 14.6 and 5.7 mg, respectively. 1.8 mg of compound **3** were isolated from fraction 7 by employing two chromatographic steps (1st, gradient: 0 min 50% B, 13 min 80% B; flow 18 mL min^−1^; UV detection at 220 nm; *t*_R_ 6.0 min. 2nd, isocratic: 45% B, flow 18 mL min^−1^; UV detection at 220 nm; *t*_R_ 7.3 min.).

**Calcaride A (6):** Pale yellow solid, [α]^22^_D_ −23.6 (*c* 0.195, MeOH), UV (MeOH) λ_max_ (log ε) 216 (4.57), 263 (4.27), 302 (3.92) nm; for 1D and 2D NMR data see Table 1; HRESIMS *m/z* 661.2503 [M + H]^+^ (calcd for C_33_H_40_O_14_, 661.2491).

**Calcaride B (7):** White solid, [α]^22^_D_ −25.3 (*c* 0.45, MeOH), UV (MeOH) λ_max_ (log ε) 216 (4.58), 263 (4.27), 302 (3.92) nm; for 1D and 2D NMR data see Table 2, Table 3, Table SI; HRESIMS *m/z* 677.2435 [M + H]^+^ (calcd for C_33_H_40_O_15_, 677.2440).

**Calcaride C (8):** White solid, [α]^22^_D_ −7.9 (*c* 0.793, MeOH), UV (MeOH) λ_max_ (log ε) 216 (4.59), 263 (4.30), 302 (3.94) nm; for 1D and 2D NMR data see Table 2, Table 3, Table SI; HRESIMS *m/z* 677.2441 [M + H]^+^ (calcd for C_33_H_40_O_15_, 677.2440).

**Calcaride D (9):** White solid, [α]^22^_D_ −71.8 (*c* 0.98, MeOH), UV (MeOH) λ_max_ (log ε) 216 (4.60), 263 (4.35), 302 (3.99) nm; for 1D and 2D NMR data see Table 3, Table 4, Table SI; HRESIMS *m/z* 693.2405 [M − H]^−^ (calcd for C_33_H_42_O_16_, 693.2400).

**Calcaride E (10):** White solid, [α]^22^_D_ −67.3 (*c* 0.05, MeOH), UV (MeOH) λ_max_ (log ε) 216 (4.61), 263 (4.35), 302 (3.98) nm; for 1D and 2D NMR data see Table 3, Table 4, Table SI; HRESIMS *m/z* 677.2467 [M − H]^−^ (calcd for C_33_H_42_O_15_, 677.2451).

The process of interconversion of **7** and **8** was studied by periodical HPLC-UV/MS analyses using the mass chromatograms for quantification of the compounds’ purities. For the experiments, purified and dried **7** and **8** were stored at 4 °C over a time period of three months.

### 3.5. Antibacterial Assays

Antibacterial assays were performed using the test strains, *Staphylococcus epidermidis* (DSM20044), *Xanthomonas campestris* (DSM 2405) and *Propionibacterium acnes* (DSM1897). *S*. *epidermidis* and *X*. *campestris* were cultivated in Trypticase soy broth (1.2% Trypticase Soy Broth, 0.5% NaCl) overnight and diluted to an OD_600_ of 0.01–0.03. 10 mM DMSO solutions of the compounds were diluted with medium to gain desired test concentrations. 10.5 µL of the compound solutions and 200 µL of the respective test strain cell suspension were transferred into a 96-well microtiter plate. Microtiter plates with *S*. *epidermidis* were incubated for 5 h at 37 °C in the dark, while microtiter plates with *X*. *campestris* were incubated for 6 h at 28 °C in the dark. After the addition of 10 µL of a resazurin solution as the detective reagent (0.2 mg mL^−1^ in phosphate-buffered saline), the incubation was continued for another 5–30 min. To evaluate cell viability, the reduction of resazurin to resorufin was determined by measuring the intensity of fluorescence at 560_Ex_/590_Em_ in a Tecan Infinite M200 plate reader.

*P*. *acnes* was grown anaerobically (Anaerocult A mini, Merck) in modified PYG medium (DSMZ medium 104) for 48 h at 37 °C in the dark. 2-day old cultures were diluted to an OD_600_ of 0.02. A volume of 200 µL of the cell suspension was transferred to a microtiter plate and mixed with 10.5 µL of the test compounds that were treated as described above. After an anaerobic incubation for 48 h at 37 °C in the dark, 10 µL of bromocresol purple (1 mg mL^−1^) were added, and the acid production of *P*. *acnes* was determined by measuring the absorbance at 590 nm (reference 690 nm) using the Infinite M200 plate reader. 

The resulting values of the antibacterial assays were compared with a positive control (10 µM chloramphenicol for *S*. *epidermidis* or *X*. *campestris* and 1 µM chloramphenicol for *P*. *acnes*) and a negative control (no compound). Minimal inhibitory concentrations (MICs) were defined as more than 20% inhibition of metabolic activity or acid production in the test strains compared to non-inhibited metabolism in the negative control.

## 4. Conclusions

Five known 15G256 components and five methylated analogs thereof were isolated from *Calcarisporium* sp. strain KF525: 15G256α, α-2, β, β-2, π (**1**–**5**) and calcarides A–E (**6**–**10**). For the first time, antibacterial properties were detected for this type of natural product. The compounds showed inhibitory bioactivities against *Staphylococcus epidermidis*, *Xanthomonas campestris* and *Propionibacterium acnes*, underlying a tight structure-activity relationship. 15G256α (**1**) displayed the strongest inhibition against *S*. *epidermidis*, with a MIC of (12.9 ± 3.6) µM, and calcaride A (**6**) exhibited the lowest MIC for *X*. *campestris* with (5.5 ± 1.3) µM. *P*. *acnes* was specifically inhibited by the compound, 15G256π (**5**), having a MIC of (14.1 ± 1.8) µM.

## Abbreviations

NMR: nuclear magnetic resonance
HRESIMS: high-resolution electrospray ionization mass spectrometry
HPLC: high-performance liquid chromatography
MS: mass spectrometry
ESIMS: electrospray ionization mass spectrometry
DEPT: distortionless enhancement by polarization transfer
COSY: correlation spectroscopy
HSQC: heteronuclear single-quantum coherence
HMBC: heteronuclear multiple bond correlation
Mult: multiplicity
ESI: electrospray ionization; calcd, calculated
SI: supplementary information
OD_600_: optical density at 600 nm
DMSO: dimethyl sulfoxide
PYG: medium, peptone yeast glucose medium
DSMZ: German collection of microorganisms and cell cultures

## Supplementary Files

Supplementary File 1 Supplementary Information (PDF, 224 KB)
