# Supplementary material for: Calcarides A–E, Antibacterial Macrocyclic and Linear Polyesters from a Calcarisporium Strain
Source: Mar Drugs. 2013 Aug 29;11(9):3309–23. doi: 10.3390/md11093309 (PMC3801122; doi:10.3390/md11093309)
Supplement: Supplementary File 1 — Supplementary Information (PDF, 224 KB) [file marinedrugs-11-03309-s001.pdf]

Supplementary Information

Figure S1. ^{1}H NMR (500 MHz, acetone-d_6) spectrum of calcaride A (**6**).
Figure S2. ^{13}C NMR (500 MHz, acetone-d_6) spectrum of calcaride A (**6**).
Figure S3. COSY (500 MHz, acetone-d_6) spectrum of calcaride A (**6**).
Figure S4. HSQC (500 MHz, acetone-d_6) spectrum of calcaride A (**6**).
Figure S5. HMBC (500 MHz, acetone-d_6) spectrum of calcaride A (**6**).
Figure S6. ^{1}H NMR (500 MHz, methanol-d_4) spectrum of calcaride B (**7**).
Figure S7. ^{1}H NMR (500 MHz, methanol-d_4) spectrum of calcaride C (**8**).
Figure S8. ^{1}H NMR (500 MHz, methanol-d_4) spectrum of calcaride D (**9**).
Figure S9. ^{1}H NMR (600 MHz, methanol-d_4) spectrum of calcaride E (**10**).
Table S1. NMR spectroscopic data (500 MHz, methanol-d_4) of calcaride B (**7**).
Table S2. NMR spectroscopic data (500 MHz, methanol-d_4) of calcaride C (**8**).
Table S3. NMR spectroscopic data (500 MHz, methanol-d_4) of calcaride D (**9**).
Table S4. NMR spectroscopic data (600 MHz, methanol-d_4) of calcaride E (**10**).

Figure S1. ^{1}H NMR (500 MHz, acetone-d_6) spectrum of calcaride A (**6**).

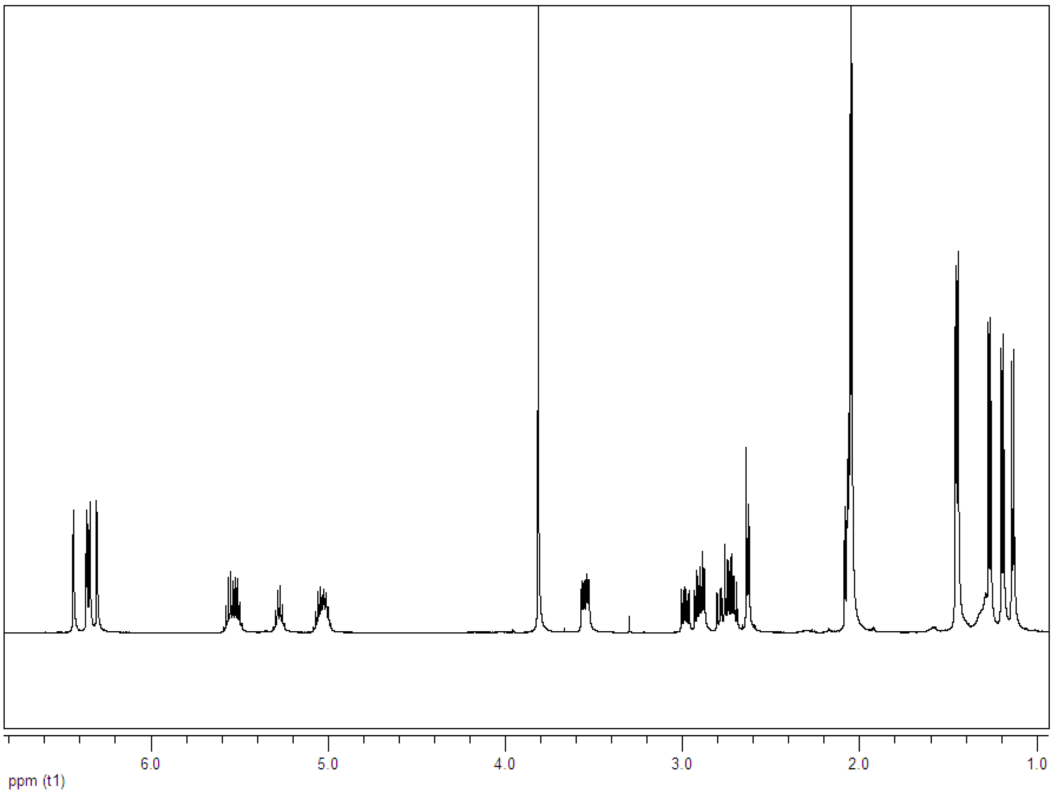

Figure S2. ^{13}C NMR (500 MHz, acetone-d_6) spectrum of calcaride A (**6**).

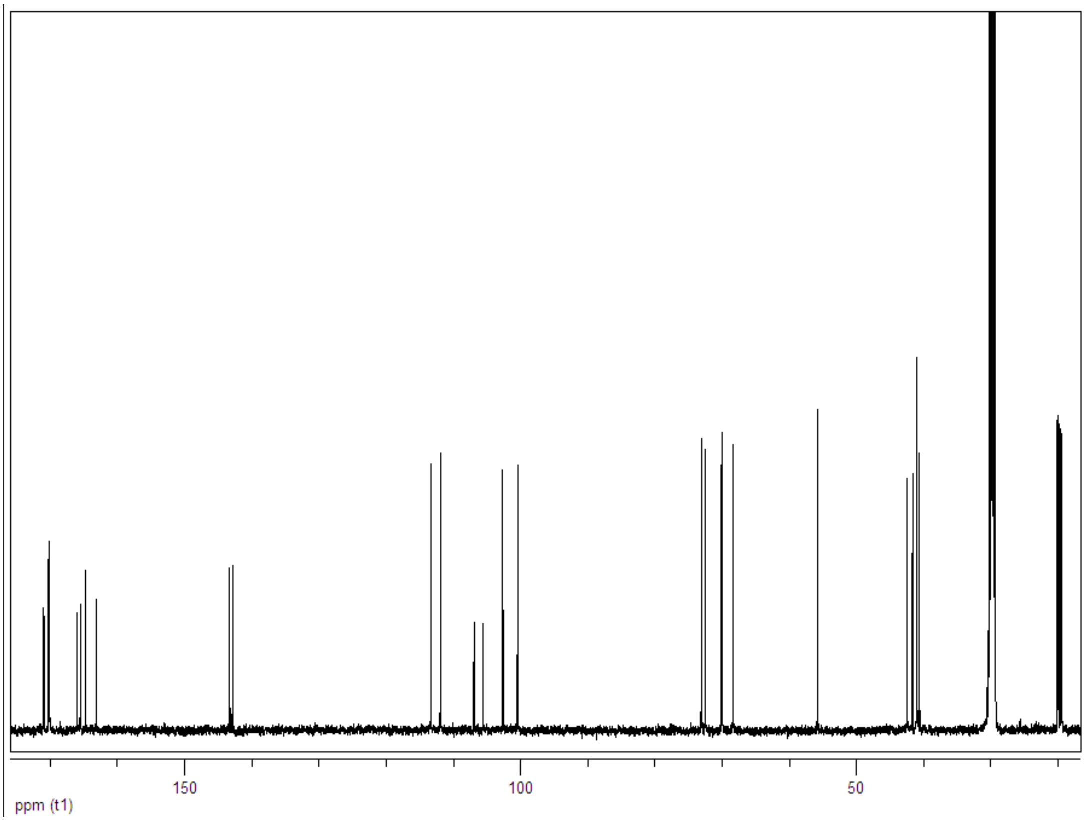

Figure S3. COSY (500 MHz, acetone-d_6) spectrum of calcaride A (**6**).

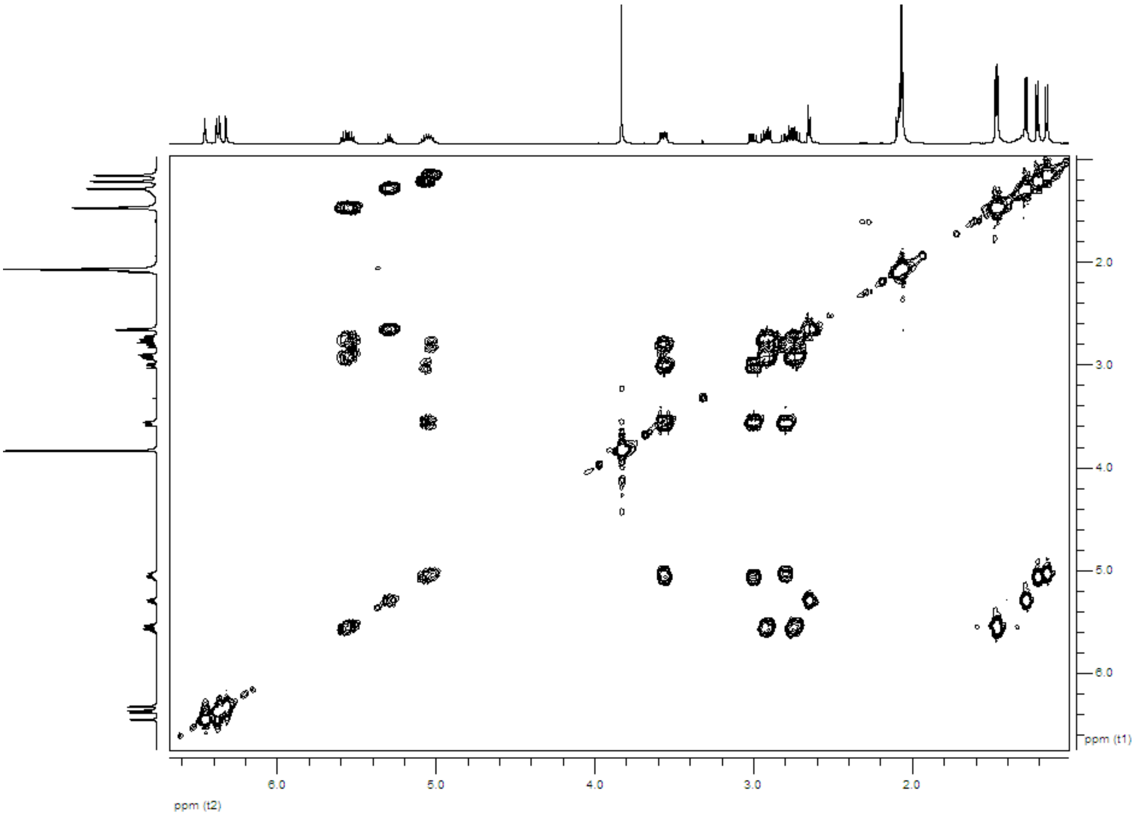

Figure S4. HSQC (500 MHz, acetone-d_6) spectrum of calcaride A (**6**).

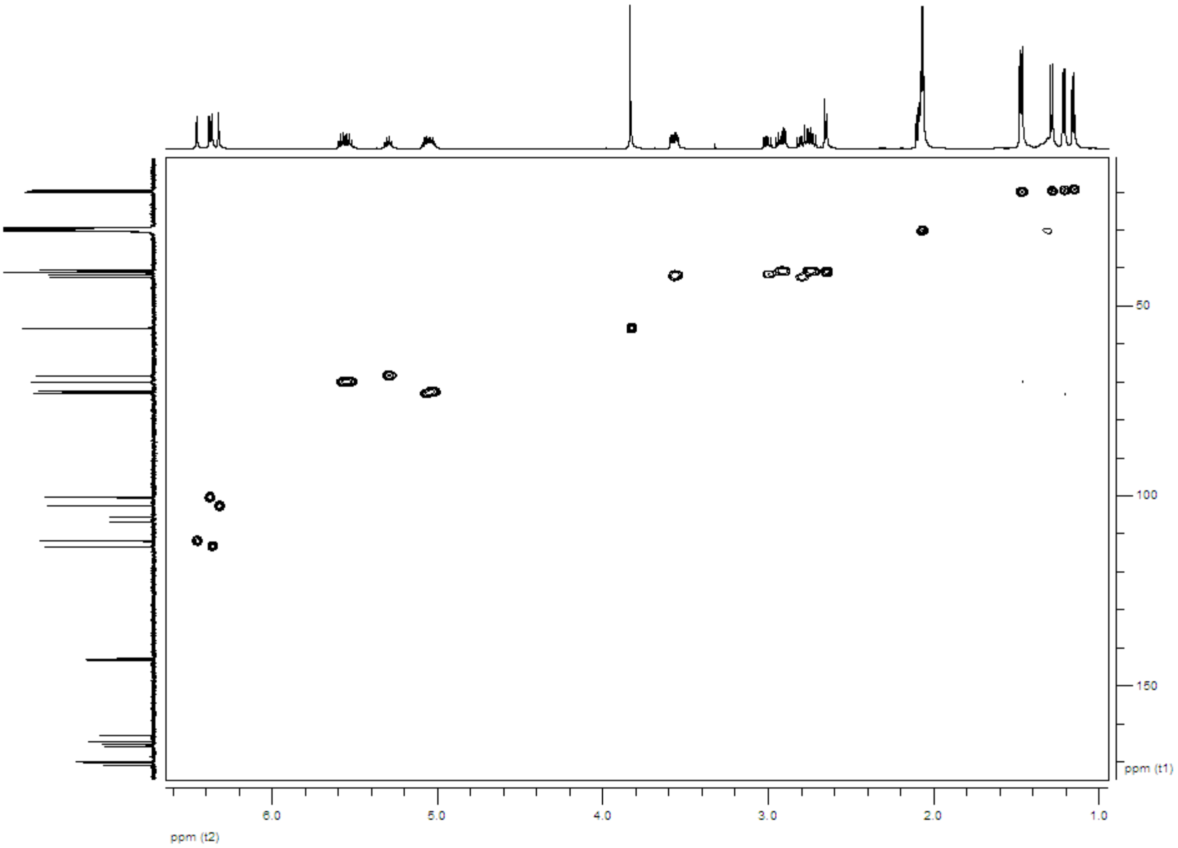

Figure S5. HMBC (500 MHz, acetone-d_6) spectrum of calcaride A (**6**).

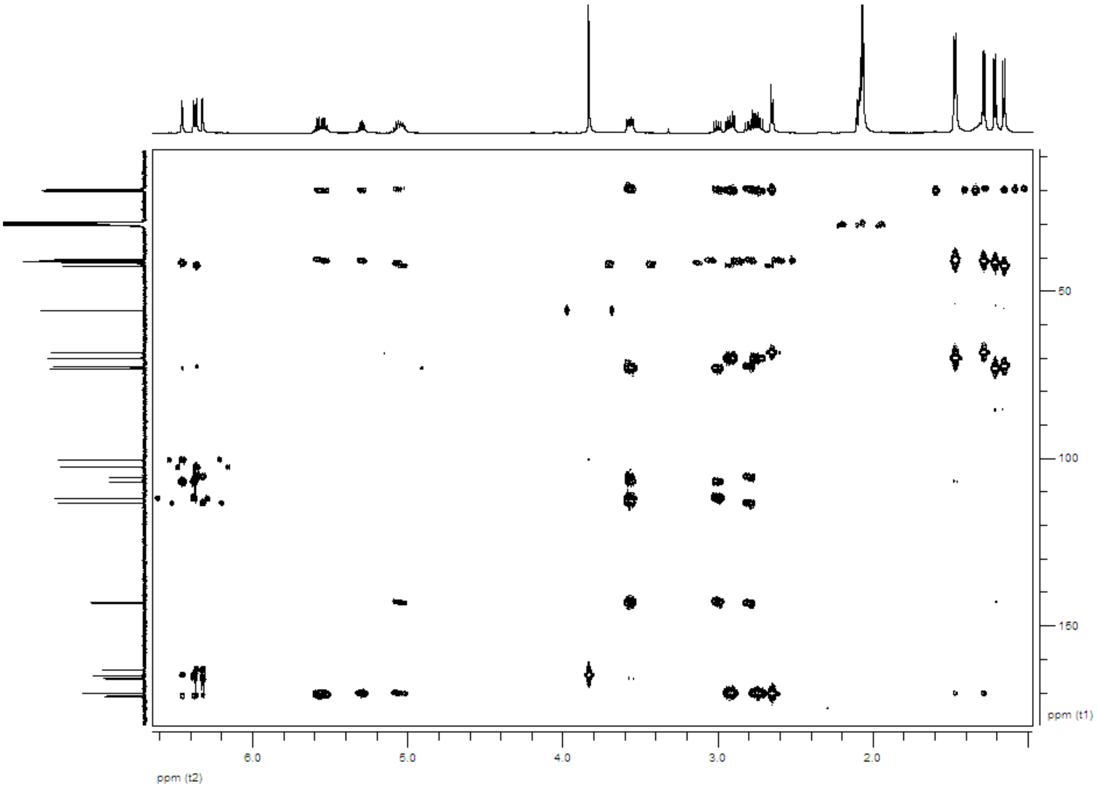

Figure S6. ^{1}H NMR (500 MHz, methanol-d_4) spectrum of calcaride B (**7**).

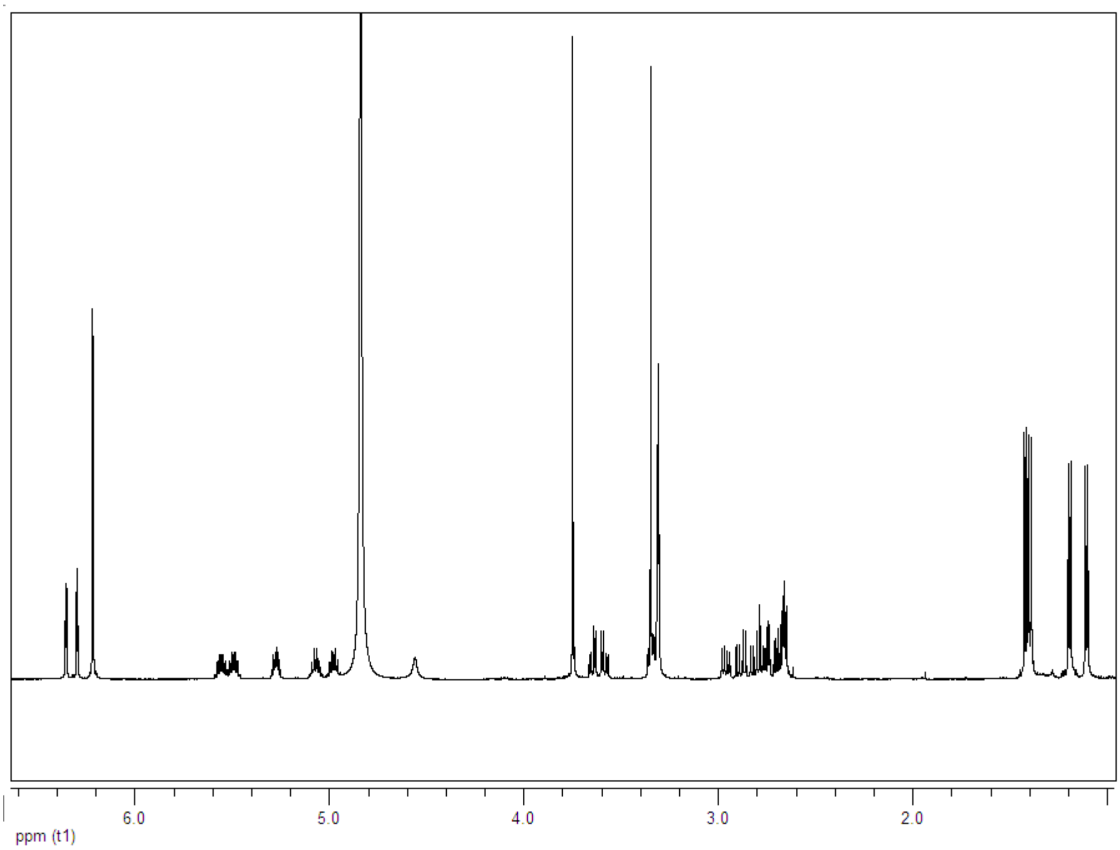

Figure S7. ^{1}H NMR (500 MHz, methanol-d_4) spectrum of calcaride C (**8**).

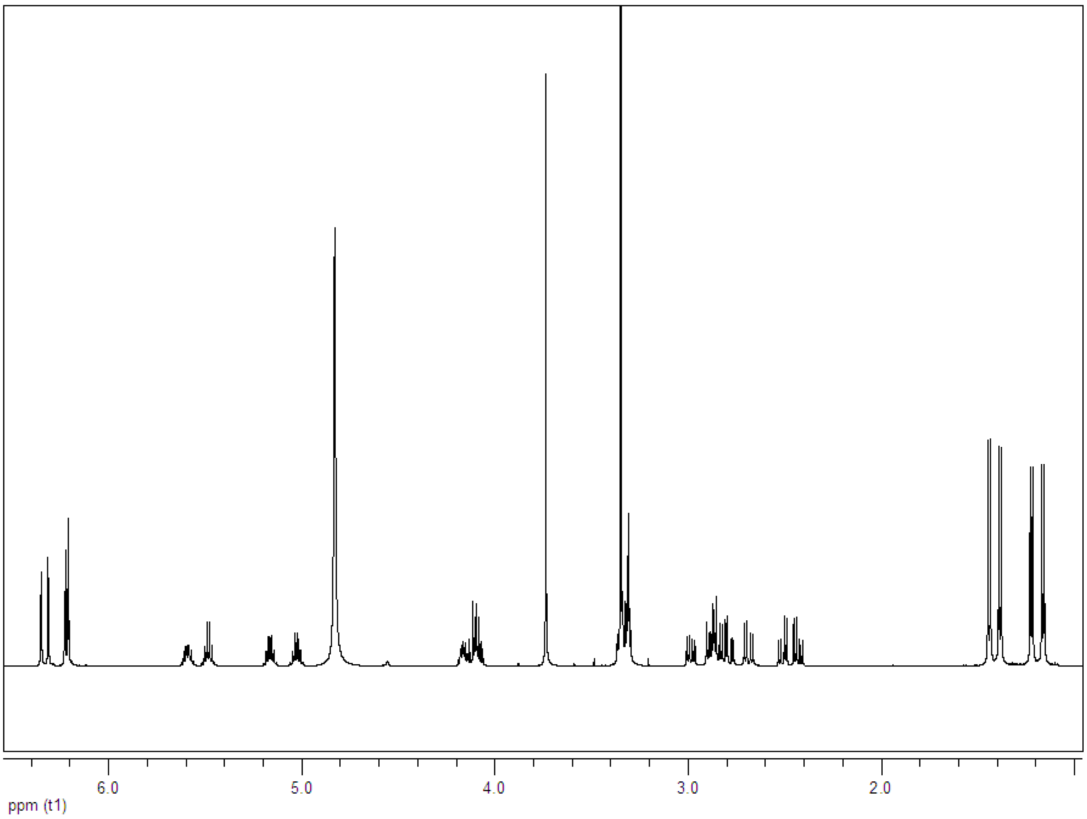

Figure S8. ^{1}H NMR (500 MHz, methanol-d_4) spectrum of calcaride D (**9**).

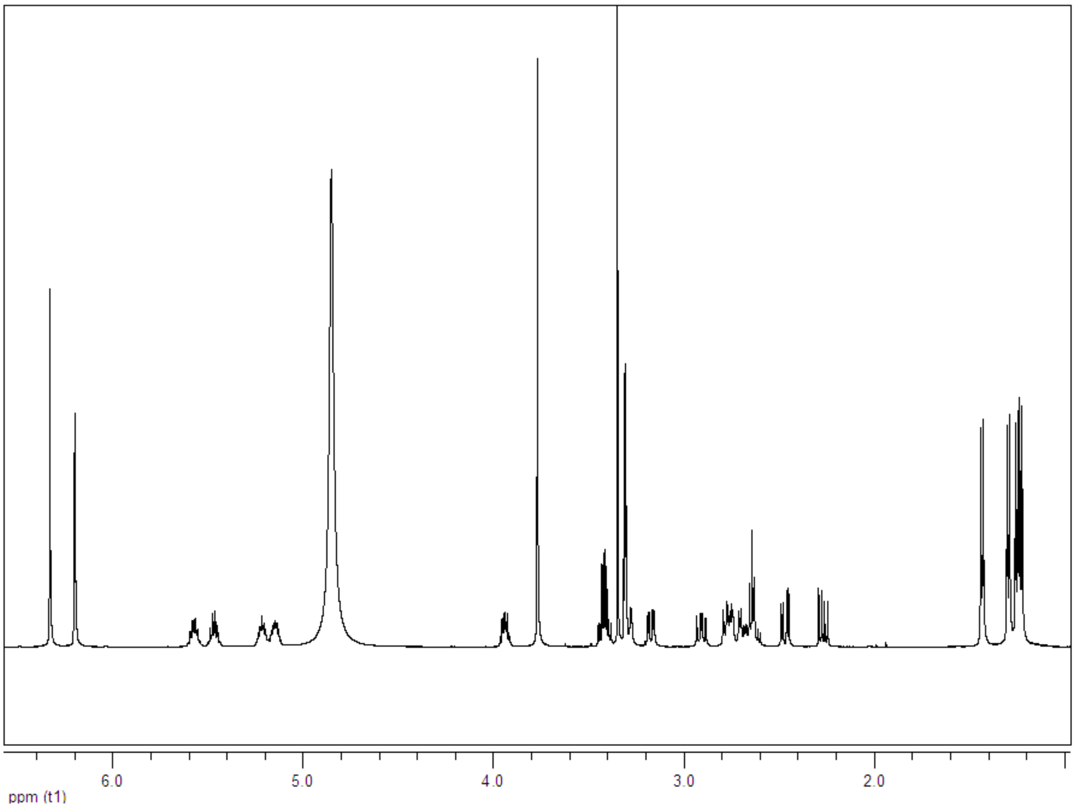

Figure S9. ^{1}H NMR (600 MHz, methanol-d_4) spectrum of calcaride E (**10**).

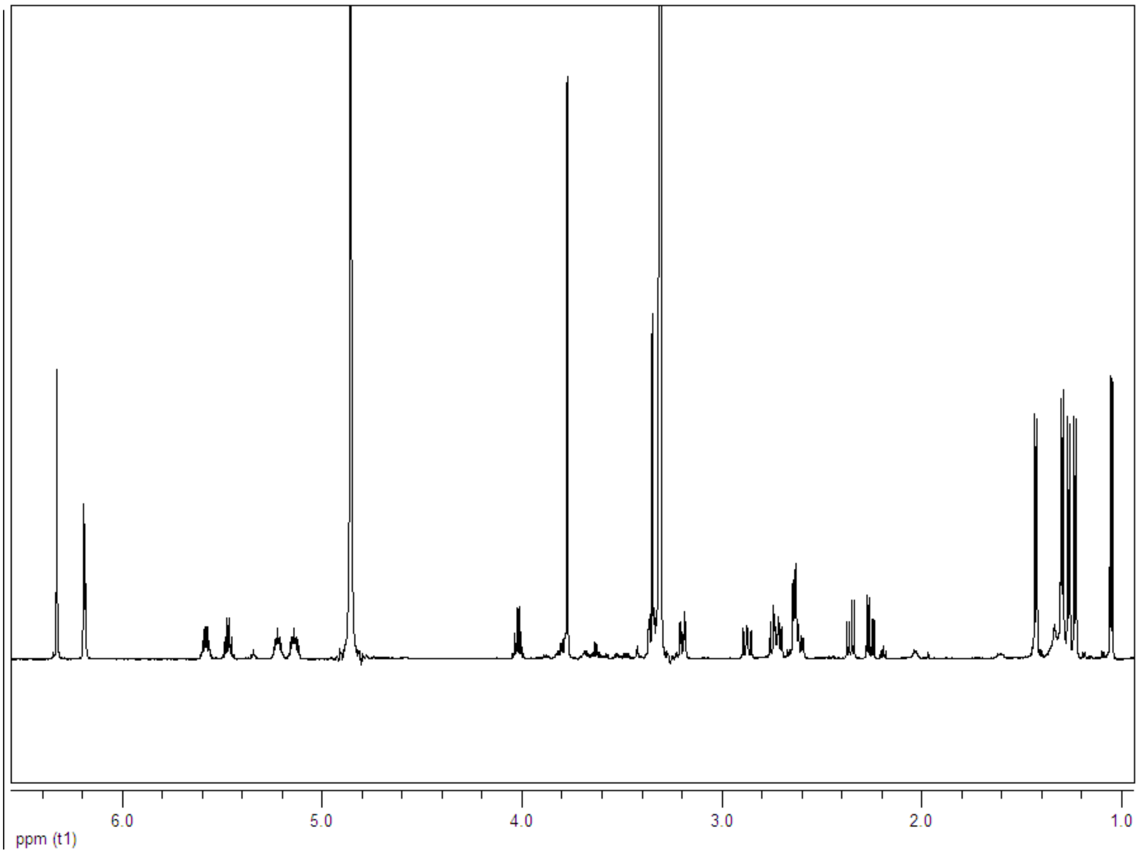

Table S1. NMR spectroscopic data (500 MHz, methanol-d_4) of calcaride B (**7**).

Position	δ_C, Type	δ_H, Mult. (J in Hz)	COSY	HMBC
1	171.5, C [a]			
2a	41.0, CH_2	2.88, dd (7.7, 16.6)	2b, 3	1, 3, 4, 5
2b		2.73, d (5.3, 16.6)	2a, 3	1, 3, 4, 5
3	70.2, CH	5.56, m	2a, 2b, 4	2, 4, 5
4	20.2, CH_3	1.42, d (6.3)	3	2, 3
5	171.13, C			
6	108.7, C			
7	164.6, C			
8	100.7, CH	6.30, d (2.6)	10	5, 6, 7, 10
9	164.9, C			
OCH_3	55.8, CH_3	3.75, s		9
10	111.5, CH	6.36, d (2.6)	8	5, 6, 8, 9, 12
11	142.6, C			
12a	41.8, CH_2	3.34 [b]	12b, 13	6, 10, 11, 13, 14
12b		2.96, dd (6.4, 13.8)	12a, 13	6, 10, 11, 13, 14
13	73.6, CH	5.07, m	12a, 12b, 14	11, 12, 14, 15
14	20.0, CH_3	1.20, d (6.2)	13	12, 13
15	171.37, C [a]			
16a	41.6, CH_2	2.81, dd (7.6, 15.8)	16b, 17	15, 17, 18
16b		2.67, dd (5.7, 15.8)	16a, 17	15, 17, 18
17	70.4, CH	5.49, m	16a, 16b, 18	16, 18, 19
18	20.2, CH_3	1.40, d (6.4)	17	16, 17
19	171.07, C			
20	106.7, C			
21	165.4, C			
22	102.8, CH	6.22, s		19, 20, 21, 23, 24, 26
23	163.5, C			
24	113.3, CH	6.22, s		19, 20, 21, 22, 23, 26, 27
25	143.2, C			
26a	42.3, CH_2	3.32 [b]	26b, 27	20, 24, 25, 27, 28
26b		2.77, dd (8.1, 13.3)	26a, 27	20, 24, 25, 27, 28
27	73.5, CH	4.98, m	26a, 26b, 28	25, 26, 29
28	19.5, CH_3	1.11, d (6.2)	27	26, 27
29	171.43, C [a]			
30a	36.7, CH_2	2.68, dd (5.1, 16.7)	30b, 31	29, 31, 32
30b		2.64, dd (8.1, 16.7)	30a, 31	29, 31, 32
31	72.7, CH	5.28, m	30a, 30b, 32a, 32b	1, 29, 30
32a	63.6, CH_2	3.65, dd (4.5, 11.9)	31, 32b	30, 31
32b		3.58, dd (5.1, 11.9)	31, 32a	30, 31

[a] Assignments of C-1, C-15 and C-29 are interchangeable; [b] Signal partially obscured and deduced from the HMBC NMR spectrum.

Table S2. NMR spectroscopic data (500 MHz, methanol-d_4) of calcaride C (**8**).

Position	δ_C, Type	δ_H, Mult. (J in Hz)	COSY	HMBC
1	171.6, C			
2a	41.2, CH_2	2.878, dd (8.1, 16.2)	2b, 3	1, 3, 4
2b		2.79, dd (4.8, 16.2)	2a, 3	1, 3, 4
3	70.5, CH	5.59, m	2a, 2b, 4	2, 4, 5
4	20.2, CH_3	1.44, d (6.4)	3	2, 3
5	171.53, C [a]			
6	108.2, C			
7	165.08, C [a]			
8	100.7, CH	6.31, d (2.6)	10	5, 6, 7, 9, 10, 12
9	165.0, C [a]			
OCH_3 [b]	55.8, CH_3	3.74, s		9 (or 7)
10	111.3, CH	6.35, d (2.6)	8	5, 6, 7, 8, 9, 12
11	142.7, C			
12a	41.7, CH_2	3.35 [c]	12b, 13	6, 10, 11, 13, 14
12b		2.99, dd (6.6, 14.0)	12a, 13	6, 10, 11, 13, 14
13	73.2, CH	5.16, m	12a, 12b, 14	11, 12, 14, 15
14	20.0, CH_3	1.22, d (6.2)	13	12, 13
15	171.3, C [a]			
16a	41.6, CH_2 [a]	2.85, dd (6.6, 16.0)	16b, 17	15, 17, 18
16b		2.69, dd (6.6, 16.0)	16a, 17	15, 17, 18
17	70.3, CH	5.48, m	16a, 16b, 18	16, 18, 19
18	20.1, CH_3	1.39, d (6.3)	17	16, 17
19	171.48, C [a]			
20	107.3, C			
21	165.14,C [a]			
22	102.7, CH	6.21, d (2.5)		19, 20, 21, 23, 24, 26
23	163.4, C			
24	112.6, CH	6.23, d (2.5)		19, 20, 21, 22, 23, 26
25	143.1, C			
26a	41.5, CH_2 [a]	3.31[c]	26b, 27	20, 24, 25, 27, 28
26b		2.885, dd (6.9, 13.4)	26a, 27	20, 24, 25, 27, 28
27	73.6, CH	5.03, m	26a, 26b, 28	25, 26, 28, 29
28	19.8, CH_3	1.16, d (6.2)	27	26, 27
29	171.9, C			
30a	40.4, CH_2	2.51, dd (6.0, 15.3)	30b, 31	29, 31, 32
30b		2.43, dd (7.2, 15.3)	30a, 31	29, 31, 32
31	67.1, CH	4.17, m	30a, 30b, 32a, 32b	29, 30, 32
32a	68.5, CH_2	4.12, dd (4.8, 11.1)	31, 32b	1, 30, 31
32b		4.08, dd (5.5, 11.1)	31, 32a	1, 30, 31

[a] Assignments of C-5, C-15 and C-19, C-7 and C-21, C-7 and C-9, C-16 and C-26, respectively are interchangeable; [b] Position of the methoxy group could not unambiguously be determined. In analogy to the other calcarides it was assumed to be linked to C-9; [c] Signal partially obscured.

Table S3. NMR spectroscopic data (500 MHz, methanol-d_4) of calcaride D (**9**).

Position	δ_C, Type	δ_H, Mult. (J in Hz)	COSY	HMBC
1	173.9, C [a]			
2a	41.8, CH_2	2.767, dd (7.5, 15.8)	2b, 3	1, 3, 4
2b		2.69, dd (5.3, 15.8)	2a, 3	1, 3, 4
3	70.8, CH	5.57, m	2a, 2b, 4	1, 2, 4, 5
4	20.1, CH_3	1.43, d (6.4)	3	2, 3
5	171.5, C [b]			
6	107.2, C			
7	166.0, C [b]			
8	100.8, CH	6.33, s		5, 6, 7, 9, 10
9	165.0, C [b]			
OCH_3	55.9, CH_3	3.77, s		9
10	112.8, CH	6.33, s		5, 6, 7, 8, 9, 12, 13
11	143.2, C			
12a	43.2, CH_2	3.29 [c]	12b, 13	6, 10, 11, 13, 14
12b		2.91, dd (9.7, 13.7)	12a, 13	6, 10, 11, 13, 14
13	73.1, CH	5.22, m	12a, 12b, 14	11, 12, 14, 15
14	20.52, CH_3	1.25, d (6.2)	13	11, 12, 13
15	171.2, C			
16a	41.8, CH_2	2.66, dd (7.2, 15.7)	16b, 17	15, 17, 18
16b		2.62, dd (5.8, 15.7)	16a, 17	15, 17, 18
17	70.0, CH	5.47, m	16a, 16b, 18	16, 18, 19
18	19.9, CH_3	1.30, d (6.4)	17	15, 16, 17
19	171.6, C [b]			
20	105.8, C			
21	166.2, C			
22	102.7, CH	6.203, d (2.6)		20, 21, 23, 24
23	163.5, C			
24	113.8, CH	6.197, d (2.6)		19, 20, 21, 22, 23, 26, 27
25	143.7, C			
26a	43.5, CH_2	3.17, dd (4.0, 13.4)	26b, 27	20, 24, 25, 27, 28
26b		2.772, dd (9.2, 13.4)	26a, 27	20, 24, 25, 27, 28
27	72.7, CH	5.15, m	26a, 26b, 28	25, 26, 28, 29
28	20.49, CH_3	1.23, d (6.2)	27	25, 26, 27
29	172.7, C			
30a	39.9, CH_2	2.47, dd (5.2, 15.4)	30b, 31	29, 31, 32
30b		2.27, dd (7.9, 15.4)	30a, 31	29, 31, 32
31	69.9, CH	3.94, m	30a, 30b, 32a, 32b	29, 30, 32
32a	66.4, CH_2	3.44, dd (4.9, 11.2)	31, 32b	30, 31
32b		3.40, dd (5.7, 11.2)	31, 32a	30, 31

[a] Signal deduced from the HMBC NMR spectrum; [b] Assignments of C-5 and C-19, C-7 and C-9, respectively are interchangeable; [c] Signal partially obscured and deduced from the HSQC NMR spectrum.

Table S4. NMR spectroscopic data (600 MHz, methanol-d_4) of calcaride E (**10**).

**Position**	**δ_C, Type**	**δ_H, Mult. (*J* in Hz)**	**COSY**	**HMBC**
1	175.9, C [a]			
2a	43.3, CH_2	2.72, dd (7.8, 15.4)	2b, 3	1, 3, 4
2b		2.61, dd (5.9, 15.4)[b]	2a, 3	1, 3, 4
3	71.5, CH	5.58, m	2a, 2b, 4	1, 2, 4, 5
4	20.3, CH_3	1.43, d (6.3)	3	2, 3
5	171.56, C [c]			
6	107.3, C			
7	166.0, C[c]			
8	100.8, CH	6.33, s		5, 6, 7, 9, 10
9	165.0, C [c]			
OCH_3	55.9, CH_3	3.77, s		9
10	112.8, CH	6.33, s		5, 6, 7, 8, 9, 12, 13
11	143.3, C			
12a	43.4, CH_2	3.35, dd (3.5, 13.6) [b]	12b, 13	6, 10, 11, 13
12b		2.87, dd (9.8, 13.6)	12a, 13	6, 10, 11, 13, 14
13	73.2, CH	5.22, m	12a, 12b, 14	11, 15
14	20.6, CH_3	1.26, d (6.3)	13	11, 12, 13
15	171.2, C			
16a	41.8, CH_2	2.65, dd (7.2, 15.6)	16b, 17	15, 17, 18
16b		2.62, dd (6.0, 15.6)	16a, 17	15, 17, 18
17	70.0, CH	5.47, m	16a, 16b, 18	15, 16, 18, 19
18	19.9, CH_3	1.30, d (6.3)	17	15, 16, 17
19	171.62, C[c]			
20	105.7, C			
21	166.3, C			
22	102.7, CH	6.19, d (2.5)		19, 21, 20, 23, 24
23	163.6, C			
24	113.9, CH	6.18, d (2.5)		20, 21, 22, 23, 26
25	143.7, C			
26a	43.6, CH_2	3.20, dd (3.8, 13.5)	26b, 27	20, 24, 25, 27, 28
26b		2.74, dd (9.5, 13.5)	26a, 27	20, 24, 25, 27, 28
27	72.6, CH	5.14, m	26a, 26b, 28	25, 29
28	20.6, CH_3	1.23, d (6.2)	27	25, 26, 27
29	172.5, C			
30a	45.1, CH_2	2.36, dd (6.9, 14.7)	30b, 31	29, 31, 32
30b		2.26, dd (6.5, 14.7)	30a, 31	29, 31, 32
31	65.4, CH	4.02, m	30a, 30b, 32	29, 30, 32
32	23.0, CH_3	1.05, d (6.2)	31	30, 31

[a] Signal deduced from the HMBC NMR spectrum; [b] Signal partially obscured; [c] Assignments of C-5 and C-19, C-7 and C-9, respectively are interchangeable.
